# The ChromaTest, a digital color contrast sensitivity analyzer, for diabetic maculopathy: a pilot study

**DOI:** 10.1186/1471-2415-8-15

**Published:** 2008-08-17

**Authors:** Roger Wong, Jaheed Khan, Temi Adewoyin, Sobha Sivaprasad, Geoffrey B Arden, Victor Chong

**Affiliations:** 1 Retinal Research Unit, Ophthalmology Department, King's College Hospital NHS Trust, Denmark Hill, London SE5 9RS, UK

## Abstract

**Background:**

To assess the ability of the Chromatest in investigating diabetic maculopathy.

**Method:**

Patients with Type 2 diabetes and no concurrent ocular pathology or previous laser photocoagulation were recruited. Visual acuities were assessed followed by colour contrast sensitivity testing of each eye using Chromatest. Dilated fundoscopy with slit lamp biomicroscopy with 78 D lens was then performed to confirm the stage of diabetic retinopathy according to the Early Treatment Diabetic Retinopathy Study.

**Results:**

150 eyes in 150 patients were recruited into this study. 35 eyes with no previous laser photocoagulation were shown to have clinically significant macular oedema (CSMO) and 115 eyes with untreated non-proliferative diabetic retinopathy (NPDR) on fundus biomicroscopy. Statistical significant difference was found between CSMO and NPDR eyes for protan colour contrast threshold (p = 0.01). Statistical significance was found between CSMO and NPDR eyes for tritan colour contrast threshold (p = 0.0002). Sensitivity and specificity for screening of CSMO using pass-fail criterion for age matched TCCT results achieved 71% (95% confidence interval: 53–85%) and 70% (95% confidence interval: 60–78%), respectively. However, threshold levels were derived using the same data set for both training and testing the effectiveness since this was the first study of NPDR using the Chromatest

**Conclusion:**

The ChromaTest is a simple, cheap, easy to use, and quick test for colour contrast sensitivity. This study did not achieve results to justify use of the Chromatest for screening, but it reinforced the changes seen in tritan colour vision in diabetic retinopathy.

## Background

The debilitating nature of untreated diabetic retinopathy promotes the need for cost-effective screening methods. Various studies have shown that cost effective screening can reduce blind registration due to diabetes [1-3]. Although seven field 30 degree stereo colour fundus photographs are the gold standard for diabetic screening, both remain relatively expensive and difficult to obtain [4,5]. In the UK, the National Screening Program for Diabetic Retinopathy utilises non-stereo digital photography as this meets the Diabetes UK standards for sensitivity and specificity.

Non-stereo fundus imaging is easier to obtain but has limitations in establishing macular oedema [6]. There is evidence that tritan colour vision is diminished in patients with diabetic maculopathy, but testing with the FM100 hue and Farnsworth-Lanthony D-15 test are labour intensive and time consuming [7]. Colour vision testing with a computer graphics system is an effective alternative [8]. This study assesses the ability of an automated, digital colour contrast sensitivity program in investigating diabetic maculopathy.

## Methods

Patients from either the Diabetic Eye Screening Service or patients returning for their follow-up appointment in the Medical Retina Service were recruited for this study. Inclusion criteria included Type 2 diabetic patients with untreated non-proliferative diabetic retinopathy (NPDR) and untreated clinically significant macular oedema (CSMO). Exclusion criteria included Type 1 diabetes, proliferative diabetic retinopathy, previous laser photocoagulation, and concurrent ocular pathology including infection, trauma, amblyopia, glaucoma, and/or vascular occlusion.

Medical history including duration of diabetes, hypertension, renal disease, recent HbA1c, and smoking were recorded. Concurrent eye disease and previous treatment were also recorded. Examination of best corrected logMar visual acuities (BCVA) was followed by colour contrast sensitivity testing of each eye by occluding the fellow eye and using the diabetic module of ChromaTest, a software program analyzing the age-corrected tritan (TCCT) and protan color contrast thresholds (PCCT). A brief explanation of what the patient is expected to see and their expected response was made prior to the test. The right eye was tested first followed by the left.

For the Chromatest, the subject is seated at a fixed distance from the monitor so the alphabetical letter displayed on the computer screen subtends a constant angle on the retina. The letter size creates an image that tests the central 6.5 degrees of the retina. The letters are displayed on a background of equiluminance. The operator has no influence on the contrast of the test letter given. The computer finds the endpoint of the test by a Modified Binary Search method; if response is correct, on the next presentation the colour difference between letter and background is halved. If response is incorrect, the colour -contrast is doubled. Incorrect responses prolong the test, but do not influence the final threshold. This method of determining thresholds leads to finite steps which reach a plateau at the colour contrast sensitivity threshold. The reproducibility of this measurement is 1%, which is the sensitivity of the test. The Chromatest has been further described in various articles [8-10]. Control data was obtained from unpublished data collected by G.B. Arden from diabetic patients without any diabetic retinopathy prior to this study (Table 1). Test and training sets are both from the group studied in this report.

**Table 1:** Colour Contrast Sensitivity in Patients with Diabetes and No Clinical Retinopathy (N = 30)

Age	Tritan	Protan
37	12.4	2.5
44	9.4	3.1
48	4.2	4.2
48	4.1	2.9
48	4.2	2.4
51	11.3	5.9
51	4.2	2.5
51	5.9	4.7
54	6.9	6.6
54	4.1	4.8
54	7.9	3.7
57	6.8	2.5
59	8.6	2.5
59	9.4	2.4
60	15.7	2.6
60	6.2	5.4
61	15.7	11.6
62	7.1	2.7
62	8.6	11.4
64	7.9	3.7
67	9.4	5.1
67	13.6	5.4
68	17.3	5.4
68	11.7	5.71
69	6.8	6.8
69	13.9	4.7
70	17.3	4.7
70	12.4	5
71	6.7	3.8
72	21.7	5.4

Control: Age, TCCT, PCCT

Dilated fundoscopy with slit lamp biomicroscopy and 78 D lens was performed by a specialist registrar (RW) to confirm the grading of CSMO according to the Early Treatment Diabetic Retinopathy Study extension of the modified Airlie House classification [11]. CSMO is defined as any retinal thickening within 500 microns of the centre of the fovea; hard, yellow exudates within 500 microns of the centre of the fovea with adjacent retinal thickening; or at least 1 disc area of retinal thickening, any part of which is within 1 disc are of the centre of the fovea.

Each age group (eg. 30–49 years old, 50–69, 70–89) separated by 2 decades was assigned pass-fail criterion for TCCT as previous data suggests age related change in threshold for tritan colour. Since this is the first study of NPDR using the Chromatest, threshold levels were derived using the same data set for both training and testing the effectiveness. Pass-fail criterion for each age group was chosen piecewise and sensitivity/specificity calculations were made according to these arbitrarily assigned levels.

Sensitivity, specificity, confidence intervals, and χ^2 ^test were calculated by web-based statistical calculator made available by Professor Lowry at Vassar College, New York . Wilcoxon Rank Sum Test for non-parametric statistical analysis was performed using web software .

## Results

150 eyes of 150 patients were included in this study. Of the 150 eyes, 115 eyes had untreated NPDR (Table 2) and 35 eyes had untreated CSMO (Table 3). Median age was 60 years. Median duration of diabetes was 16.0 years.

**Table 2:** Colour Contrast Sensitivity in Patients with NPDR (N = 115)

Age	Log Mar VA	Tritan	Protan
31	0	13.6	3.4
32	0	5.2	3.2
32	0	6.7	2
32	0.2	15.4	3.2
41	0	16.1	15.4
41	0	6.1	2.1
41	0	6.2	2.1
41	0	6	1.7
41	0	8.4	3.9
42	0	11.4	3
44	0.2	9.6	4.8
44	0.2	13.3	8.1
45	0.2	16.1	4.2
45	0.2	22.1	5.5
45	0.4	19.9	5.8
48	0	5.6	2.9
48	0.5	20.6	3.8
48	0.6	29.5	5
49	0	7.4	3.4
49	0	6.3	2.2
49	0	8.4	3.9
49	0	8.4	2.6
49	0	9.4	3.1
49	0	9.9	3.4
49	0	10.3	2.9
49	0	30.5	6.1
49	0	34.5	4
49	0.1	33.6	6
49	0.7	9.2	2.6
49	0.7	12.2	3.6
51	0	13.6	4.4
51	0.1	18	5.8
51	0.2	19.1	7
52	0	10.8	2.6
52	0.2	82.4	9.3
54	0	9	3.1
54	0	22.1	4.6
54	0.2	23.6	4.3
55	0	14.4	3.1
55	0	20.2	5.4
55	0.2	18.4	3.5
55	0.2	17.6	2.1
55	0.3	19.6	4.4
55	0.3	85.9	7.7
55	0.4	22.1	7.7
56	0	8.1	2.7
56	0	11.1	2.5
56	0.1	6.6	2.6
57	0	10.3	3.6
57	0.1	6.7	2.9
57	0.1	7.2	2.1
57	0.2	14.9	2.9
58	0.1	13.9	3.8
58	0.2	11	3.3
58	0.2	21.4	2.8
58	0.2	38	3.8
59	0.2	6.8	2.1
59	0.2	6.3	1.4
59	0.2	10.1	2.7
60	0.2	8	3.1
60	0.2	12.2	4.4
61	0	5.7	2.7
61	0	7.5	2.5
61	0.2	8.6	2.7
61	0.2	13.4	2.8
62	0	10.4	2.8
62	0.3	98.7	78.2
62	0.3	98.7	75.7
63	0	9.9	4
63	0.1	15.4	5
63	0.1	25.3	6.5
64	0	18.5	3.7
64	0.2	20.2	4
64	0.2	75.7	21.4
65	0.3	15.4	6.3
65	0.3	37.9	19.9
67	0	18.3	7.7
67	0	20.6	6.7
67	0.1	19.9	4.6
67	0.1	57.7	3.8
67	0.2	8.1	2.5
67	0.3	20	6.5
67	0.3	50.4	2.9
67	0.5	52.4	8.4
67	0.6	18.1	6.7
68	0.1	32.7	6
68	0.2	10.6	2.7
68	0.2	31.5	3.9
69	0	14.4	4.4
69	0.1	49.6	6.2
69	0.5	19.9	5.2
71	0	9.2	13.3
71	0	11.1	3.8
71	0.1	7.2	13.7
71	0.2	9.6	2.5
72	0.2	21.5	5.7
72	0.4	5.5	2.6
72	0.4	60.3	6.1
72	0.5	34.8	6.4
72	0.6	18.6	3.3
75	0	12.9	2.2
75	0.1	19.9	4
75	0.3	40.4	3.6
76	0.3	27.6	4.4
76	0.3	70.5	9.6
77	0.1	11.9	3.6
78	0	24	5.2
78	0.2	17.6	4
78	0.2	20.9	7.1
78	0.3	22.4	12.9
79	0.5	52.6	21.7
79	0.5	98.7	67.6
82	0	13.5	5.2
82	0.2	23.6	6.8

NPDR patients: Age, VA, TCCT, PCCT

**Table 3:** Colour Contrast Sensitivity in Patients with CSMO (N = 35)

Age	LogMar VA	Tritan	Protan
31	0	8.5	3.6
31	0	11.1	4
42	0.2	14.1	4.5
44	0	7	1.9
44	0	18.8	2.6
51	0.2	8.8	2.6
52	0	29.6	3.5
52	0.3	72.3	10.7
55	0.2	18.4	3.5
56	0.3	18.4	2.9
56	0.5	36	5.6
58	0.1	7.7	2.7
58	0.3	78.2	13.7
59	0.2	23.6	3
62	0	70.5	7.7
62	0.1	49.9	11.4
63	0.4	27.3	6.7
65	0.1	85.9	14.4
65	0.3	98.7	16.9
67	0.1	16.1	3.2
67	0.2	11.8	3
67	0.3	80.8	12.4
68	0.2	13.3	3.2
69	0.1	23.3	5.3
69	0.5	30.3	16.1
70	0	21.5	6.8
70	0	35.4	5.6
70	0	32.7	5.5
70	0	62.8	9
70	0.5	98.7	20.8
71	0	98.7	14.7
71	0.2	64.8	20
71	0.3	98.7	42.3
72	0.7	68	18.4
72	0.9	57.7	16.9

CSMO patients: Age, VA, TCCT, PCCT

Median LogMar BCVA for NPDR patients was 0.20 and for CSMO patients was 0.20. Interquartile range for VA NPDR and CSMO was 0.20 and 0.30, respectively. Median PCCT for NPDR was 3.9% and for CSMO patients was 5.6%. Wilcoxon Rank Sum Test analysis revealed statistical significant difference between CSMO and NPDR eyes for PCCT (p = 0.01). When compared to controls with sample size N = 30 (Table 1), PCCT for NPDR had no statistical significance (p = 0.15) whereas PCCT for CSMO was significant (p = 0.002). Median TCCT for NPDR was 15.4% and for CSME patients was 29.6%. Statistical significance was found between CSMO and NPDR eyes for TCCT (p = 0.0002). Both were also statistically significant when compared to controls (p < 0.001)

The piecewise pass/fail criterion for TCCT for each age group was as follows: 11.0 (30–49 year old); 23.0 (50–69 year old); 32.0 (70–89 year old). Sensitivity and specificity for screening of CSMO using the above pass-fail criterion for age matched TCCT results achieved 71% (95% confidence interval: 53–85%) and 70% (95% confidence interval: 60–78%), respectively (Table 4).

**Table 4:** χ^2 ^test for TCCT detection of CSMO

	True Positive	True Negative	Total
Test Positive	25	35	60
Test Negative	10	80	90

Total	35	115	150

Sensitivity = 71% (CI: 53–85%), Specificity: 70% (CI: 60–78%); χ^2 ^test: p < 0.0001 comparing proportions of true positives among the test positive versus test negative subjects

When repeating the analysis in Table 4 for only subjects with logMar BCVA > = 0.1, sensitivity to detect CSMO improves to 75% (CI: 47–91%) and specificity to 85% (CI: 67–89%) p = 0.0002. Similarly, when repeating the analysis in Table 4 for only subjects with CSMO with central macular thickening, sensitivity to detect CSMO improves to 83.3% (CI: 58–96%) p < 0.0001.

## Discussion

Cost effective screening for chronic and debilitating disorders such as diabetic retinopathy is not only important to the well being of the patient, but these healthy adults contribute to the economy of a nation. With the rise in type 2 diabetes in obese adolescents due to dietary and lifestyle changes, the need for an optimal method of screening for sight threatening diabetic retinopathy becomes a critical essential [12].

Abnormal protan and especially tritan colour vision is associated with diabetic retinopathy [13]. Blue-yellow defect has also been described in both diabetic retinopathy and glaucoma [14]. In contrast to the optotype used for testing macular function, the Chromatest has a separate glaucoma module for which it is designed to measure peripheral colour sensitivity changes in an arcuate manner using a central fixation point. This study did not cross examine patients with glaucoma and diabetic retinopathy using both glaucoma and macular modules, but it is feasible that further testing may reveal an overlap in colour defect for these patients. Although the mechanism of altered colour vision is unknown, there is evidence that reduced retinal oxygen saturation is associated with impaired colour vision in diabetics [15]. Error scores in colour vision have been found to be directly correlated to severity of macular oedema [16]. This may be similar to the effects of retinal detachment where photoreceptors are shifted obliquely [16]. Correlation between selective loss of short wavelength pathway sensitivity and the severity of diabetic macular oedema has been demonstrated [17,18]. Therefore, we have concentrated on the study of untreated CSMO to ascertain the viability of such a screening method. The use of smaller letters (1.5 degree; Chromatest module for age related macular degeneration) might give better results for CSMO as it may test macular function better than the larger 6.5 degree optotype.

This study included only patients with type 2 diabetes to reduce the possible variability in pathogenesis. Although the mechanism of diabetic retinopathy is likely to be identical in both type 1 and type 2 diabetes, previous studies such as the Early Treatment Diabetic Retinopathy Study and Diabetic Retinopathy Study have investigated each type of diabetes separately. Laser photocoagulation was an exclusion criterion as it affects tritan colour vision [19]. Cataract and pseudophakia were not excluded as both are more common in diabetics and exclusion would have limited the usefulness of the Chromatest in screening. It is understood that lens-yellowing effects due to cataract may cause pre-retinal absorption of short-wavelength light resulting in tritan deficits. This may have influenced the overall sensitivity and specificity of the study, but it was a representation of the realistic setting clinicians experience in their practice.

In colour contrast testing, the higher the TCCT or PCCT score, the more abnormal the result compared to age-matched normal levels. 30% (35 of 115) patients with NPDR had TCCT above normal levels. 12 male patients were suspected to have congenital colour blindness as their PCCT were considerably worse than normal and not corresponding to their visual acuity or their fundus appearance. This was not confirmed with any other mode of investigation as the study was aimed at mimicking realistic clinical setting where high volume testing can be conducted without further time consuming tests. 16 cases had severe NPDR and may have contributed to the poor results whereas the remaining 7 had results not corresponding to their fundus appearance. We postulate that these 7 eyes may have had concurrent disease indistinguishable by indirect biomicroscopy such as more advanced ischaemia. Ultimately, fluorescein angiography may have further elucidated the true pathology.

29% (10 of 35) CSMO patients had TCCT better than normal levels. 8 eyes had CSMO qualified as 1 disc area of retinal thickening within 1 disc area of the fovea. 2 eyes had exudates with associated retinal thickening within 500 microns of the fovea, but both were left eyes and it is possible that the patients were able to perform educated guesses because they had been conditioned following testing with their right eye.

Unfortunately, we were forced to obtain normal threshold levels through the same dataset. These levels were obtained through analysis of cases without CSMO. Therefore, the results may be biased. However, because this device is relatively new and the limited availability of further data from diabetics, we are limited to using this dataset to obtain "normal" threshold values. Further data will strengthen our case of the power of this diagnostic tool.

The Chromatest is unable to successfully screen those patients with congenital blindness and performs less well for patients without foveal pathology. Conditioning following testing with the right eye may also allow patients to perform better on their left eye. From anecdotal evidence, time for testing of the second eye was observed by the investigators to be shorter than the first eye. Repeated testing which was not done in our study may alleviate this problem. This study has studied more untreated CSMO eyes with colour vision than any other that have been published, but it requires more data to solidify our findings. Colour contrast analysis may become a useful tool for defining the need for laser treatment, but so far our experience fails the Exeter Standards of the British Diabetic Association (Diabetes UK), which established screening levels of at least 80% sensitivity and 95% specificity [20].

Despite the limitations of the results, there was no discrimination for age and visual acuity due to the ease of the test. All patients were able to perform this test unlike the 1.5% of patients failing to perform another automated TCCT test [21]. Average test time was fast at 5 minutes and requires no mydriasis unlike fluorescein angiography and fundus photography. Conditioning after repeated testing is an issue for reliability, but this study was aimed at mimicking realistic clinical settings where patients have no experience of colour contrast testing. Further studies to distinguish repeatability and data for classifying normal results from abnormals are planned. The equipment required is relatively cheap and readily available compared to those required for optical coherence tomography or stereomacular photographs. It is also a non-invasive procedure and less labour intensive compared to fluorescein angiography.

## Conclusion

Non-ophthalmic doctors can have a retinopathy detection rate of 49% compared to 96% for ophthalmologists [22]. Therefore, a cost effective method for screening is essential for diabetic retinopathy. Screening by digital photography proposed under the National Service Framework is offered to all patients with diabetes in the United Kingdom. It is supplemented by biomicroscopy by the ophthalmologists in monitoring and treating sight threatening disease. Furthermore, optical coherence tomography has become a powerful tool in screening and monitoring CSMO with sensitivity and specificity rates of near 80% and 90%, respectively [23]. Perhaps with further investigation, TCCT testing may become a supplement for detecting and monitoring sight threatening pathology without much equipment or trained technicians. However, with current data, all forms of TCCT testing including the Chromatest do not qualify for use in screening for CSMO.

## Competing interests

The authors declare that they have no competing interests.

## Authors' contributions

RW examined patients, conducted investigation, conceived, drafted the manuscript. TA performed the statistical analysis. JK compiled patient list and conducted investigation. SS compiled patient list and conducted investigation. GA performed the statistical analysis. VC conceived of the study, and participated in its design and coordination. All authors read and approved the final manuscript.

## Pre-publication history

The pre-publication history for this paper can be accessed here:


